# Die Bedeutung von Gratifikationen bei der Aneignung neuer Medien im höheren Lebensalter: Ergebnisse einer qualitativen Studie mit hochaltrigen Nutzerinnen und Nutzern digitaler Medien

**DOI:** 10.1007/s00391-020-01833-z

**Published:** 2020-12-28

**Authors:** Julian Wangler, Michael Jansky

**Affiliations:** Zentrum für Allgemeinmedizin und Geriatrie, Universitätsmedizin Mainz, Am Pulverturm 13, 55131 Mainz, Deutschland

**Keywords:** Mediennutzung, Medienaneignung, Neue Medien, Digitalisierung, Internet, Ältere Menschen, Alter, Media usage, Media adoption, New media, Digitalization, Internet, Older people, Old age

## Abstract

**Hintergrund:**

Eine verbreitete Annahme geht davon aus, dass Menschen im Laufe der Jugend und des Erwachsenenlebens bestimmte Dispositionen des Umgangs mit Medien erlernen, die es ihnen später erschweren, neue mediale Nutzungsmuster zu entwickeln. Jenseits theoretischer Annahmen besteht bislang ein Mangel an explorierenden Untersuchungen, die der Frage nachgehen, unter welchen Bedingungen ältere Menschen digitale, ihnen bislang nicht vertraute Medien aufgreifen und in ihren Alltag einbeziehen.

**Material und Methoden:**

Zwischen Oktober 2019 und März 2020 wurden 16 halbstandardisierte Einzelinterviews mit Personen zwischen 80 und 92 Jahren geführt, die sich in den letzten Jahren ein digitales Medium neu erschlossen und in ihren Alltag integriert haben.

**Ergebnisse:**

Die Interviewten haben sich neue Medien zielgerichtet angeeignet. Dabei fällt auf, dass Aneignungsprozesse maßgeblich durch gravierende Veränderungen der Lebensumstände angestoßen wurden. Die Intention der Aneignung eines internetfähigen Mediums lag bei den meisten Interviewten im Wunsch begründet, wenige ausgewählte Funktionen nutzen zu wollen. Erst mit einiger Verzögerung sind neue Möglichkeiten der Onlinenutzung ausgekundschaftet worden. Im Hinblick auf die Motive und Gratifikationen der Aneignung wurden folgende Muster identifiziert: Neue Medien als … 1) Hobbyerweiterung, 2) Hilfsnetz, 3) Kompensationsinstrument, 4) Anschlussmöglichkeit, 5) Ausbruch aus dem Alltag.

**Diskussion:**

Soweit gewinnbringende, alltagsnahe Nutzungspotenziale wahrgenommen werden, zeigen ältere Menschen ein hohes Maß an Anpassungsfähigkeit in Bezug auf neue Medien. Vor diesem Hintergrund erscheint ein gratifikationsorientiertes Modell als vielversprechender Ausgangspunkt, um Voraussetzungen für die Medienaneignung anhand von Motiven zu erklären, die sinnhafte Anreize schaffen, den Umgang mit neuen Medien im höheren Lebensalter zu erlernen.

## Abstract (en)

**Background:**

A widespread assumption is that people learn certain ways of dealing with media in the course of their youth and adult life, which later make it difficult for them to develop new patterns of media usage. Beyond theoretical assumptions, there is a lack of exploratory studies that investigate the conditions under which older people pick up new media technologies and integrate them into their everyday lives.

**Material and methods:**

From October 2019 to March 2020 a total of 16 semi-standardized individual interviews were carried out with people aged between 80 and 92 years. The interviewees had learned to use a digital medium on their own in the past few years and had integrated this into their everyday lives.

**Results:**

The interviewees appropriated new media in a targeted manner. It is noticeable that processes of appropriation were largely triggered by severe changes in living conditions. In addition, most of the interviewees learned how to use a digital medium because they wanted to use specific functions. With some delay, new options for online use have been explored. With respect to the primary motivations and gratifications of appropriation, the following patterns were identified: new media as 1) hobby expansion, 2) support network, 3) compensation instrument, 4) connectivity option, 5) breaking out of everyday life.

**Conclusion:**

As far as older people perceive practical everyday potentials of use, they show a high degree of adaptability with respect to new media. Against this backdrop, a gratification-oriented model appears to be a promising starting point for explaining the requirements for media appropriation on the basis of motifs that create meaningful incentives to learn how to use new media in old age.

Eine gängige Annahme lautet, dass Menschen bestimmte Sozialisationsprozesse in Bezug auf den Umgang mit Medien durchlaufen. Aus dieser Perspektive stellt es für ältere Menschen eine Herausforderung dar, die Anwendung neuer Medien zu erlernen und diese in ihren Lebenskontext zu integrieren. Im Zuge einer qualitativen Studie wurden Personen zwischen 80 und 92 Jahren interviewt, die sich digitale Medien selbstständig angeeignet haben. Entscheidend war das Vorhandensein spezifischer Motive, die sinnhafte Anreize stiften, neue Medien dauerhaft zu nutzen.

## Hintergrund

Im Vergleich mit anderen Rezipientengruppen weisen ältere Menschen – insbesondere in der Altersgruppe ab 70 Jahre – eine höhere Nutzungsintensität linearer Medien wie Fernsehen und Zeitung auf, sind stärker an stabilen Routinen der Medienrezeption orientiert und nutzen digitale Angebote wie Computer, Internet oder Smartphone seltener [7, 10, 17–19, 27, 28, 31]. Einige Autoren schlussfolgern daher, dass der digitale Medienumbruch „noch lange nicht im gleichen Ausmaß wie bei den jüngeren Alterskohorten angekommen“ sei [6, S. 147]. Um dies zu erklären, zieht die Alter(n)smedienforschung verschiedene Theorien heran [20].

Aus einer ökonomisch-strukturalistischen Perspektive betrachtet, basiert die Bereitschaft zur Aneignung neuer Technologien auf einer Kosten-Nutzung-Kalkulation, die die subjektiv wahrgenommene Nützlichkeit sowie die Aneignungsschwierigkeit gegeneinander abwägt [9, 29].

Im Feld kulturalistischer Ansätze legt das auf Mannheim aufbauende „Mediengenerationen“-Konzept [1, 24] die Annahme zugrunde, dass über Sozialisationsprozesse fundamentale Lern‑, Aneignungs- und Handlungsprozesse in Bezug auf Medien geprägt werden [22]. Die erworbenen Dispositionen in Bezug auf eine bestimmte „(Leit)Medientechnik“ [30, S. 37] bleiben aufgrund der biografisch erworbenen Erfahrungen und Gewohnheiten im Laufe des Lebens weitgehend stabil und begünstigen „spezifische Wahrnehmungs- und Umgangsweisen“ bei der medialen Rezeption und Nutzung [30, S. 33]. Falls es bei Personen, die analoge Medien gewohnt sind, zur Aneignung digitaler Angebote kommt, kann unterstellt werden, dass das mediennutzende Subjekt solche „mit einer impliziten Handlungslogik adaptiert, die sich an Erfahrungen mit analogen Medien orientiert“ [34, S. 42].

Eine andere Betrachtung lässt sich im Rekurs auf Bourdieu unter dem Begriff des „medialen Habitus“ subsumieren [2, 5]. Hier wird ein lebenslanger Prozess der Psychogenese mit Blick auf die Herausbildung alltäglicher Handlungsmuster, Praxisformen und Verhaltensstrategien fokussiert. Trotz ausgeprägter Stabilität werden diese habituellen Muster der Lebensführung als wandelbar erachtet, wenn sich Lebenskontexte ändern [13, 36]. Demgemäß kann bezweifelt werden, „ob Menschen im Laufe ihrer Biografie tatsächlich ein relativ fixes Repertoire an medialen Handlungslogiken erwerben“ [22, S. 54]. Bei Veränderungen von Lebenslagen und -umständen denkbar, dass sich im Alter der mediale Habitus verändert [2, 13]. Infolgedessen können auch die Einstellung zu bestimmten medialen Angeboten sowie die Bereitschaft zu deren Nutzung Umbrüche erfahren.

Eine sozialwissenschaftliche Theorie, die Mediennutzung als hoch individuell und differenziell modelliert, ist der ursprünglich von Blumler und Katz konzipierte „Uses-and-gratifications“-Ansatz [4, 33]. Ihm zufolge ist Medienhandeln Folge von absichtsgeleiteten Handlungen, die zielgerichtet und selektiv sind und sich aus dem persönlichen Alltag ergeben [12, 37]. Soziodemografische Variablen wie Alter, Geschlecht, Bildung etc. stellen hier nur eine Komponente dar. Neben situationsbezogenen Kontexten sind weitere persönlichkeitsbezogene Aspekte (z. B. Werte, Überzeugungen, Erfahrungen, soziale Netzwerke) zu berücksichtigen. Dieser komplexen Ursprungslage folgen Bedürfnisse als generelles Mangelgefühl bzw. Motive als individuelle Problemlagen, für welche die Mediennutzung als Problemlösungsstrategie dienen kann [33]. In der stark heterogenen Uses-and-gratifications-Forschung werden Motive eher pragmatisch als Ausgangspunkt für wahrgenommene Nutzungszwecke sowie als alltagsnahe, sinnstiftende Anreize definiert, die dann in Gratifikationen, also einer Erfüllung von Nutzungsabsichten und -zielen, münden können [12]. Daran orientiert sich der vorliegende Beitrag.

Im höheren Lebensalter ist anzunehmen, dass die Motive zur (Nicht‑)Nutzung bestimmter Medien mit einem dynamischen Wechselspiel aus Entwicklungsverlusten bzw. -gewinnen sowie persönlichen Bewältigungs- und Umgangsweisen mit dem Alter(n) interagieren [12, 25, 38]. Verbinden ältere Menschen einen sinnhaften und alltagdienlichen Nutzen mit neuen Medien (z. B. erweiterte Teilhabemöglichkeiten), fällt die subjektiv erlebte Hürde einer Aneignung geringer aus [7, 21].

Jenseits theoretischer Annahmen und quantitativer Studien zur Mediennutzung (u. a. [3, 7, 11, 17, 31]) fehlt es an empirischen Befunden, die den „Stellenwert des Mediums in den alltäglichen Lebenswelten“ explorieren [14, S. 41] und auf dieser Grundlage zu bestimmen versuchen, unter welchen Voraussetzungen es im höheren Lebensalter ab 70 Jahre zur Hinwendung zu neuen Medien kommt, und welche Spezifika diese Nutzung aufweist. Dabei sind Anlässe und Motive im Prozess der Medienaneignung in den Blick zu nehmen [11].

## Methodik

Die auf halbstandardisierten Interviews basierende Studie verfolgte das Ziel zu beleuchten, unter welchen Bedingungen ältere Menschen digitale, ihnen bislang nicht vertraute Medien aufgreifen. Das Erkenntnisinteresse bündelte sich in folgenden Fragestellungen:

- Was war der Anlass bzw. die Nutzungsabsicht, sich das digitale Medium anzueignen?
- Wie bzw. zu welchem Zweck (Nutzungsziele) wird das digitale Medium eingesetzt?
- Welcher Nutzen wird subjektiv wahrgenommen?
- Wie hat sich der Alltag verändert, seit das Medienangebot genutzt wird?
- Was sagen die Erkenntnisse darüber aus, wie ältere Menschen sich digitale Medien aneignen?

### Leitfaden und Rekrutierung

Entlang der Fragestellungen wurde ein kompakter Leitfaden erstellt. Dieser wurde sowohl deduktiv unter Berücksichtigung bestehender Vorstudien (v. a. [8, 14, 15, 39]) als auch induktiv im Zuge erster Gespräche abgeleitet.

Rekrutiert werden sollten ältere Personen, die sich in den letzten Jahren ein digitales Medium von Grund auf neu erschlossen haben, ohne dass sie vorher einen Bezug zu digitalen Medien hatten. Es erfolgte eine Konzentration auf Personen ab 80 Jahre, da vermutet werden kann, dass die subjektiv empfundenen Hürden für eine Adaptierung neuer Medien in dieser Altersklasse höher liegen [11].

### Sample

Neun Interviewpartner wurden über den entfernten Bekanntenkreis der Autoren gewonnen, 7 Personen über einen Aufruf in 3 großen Internetforen für Senioren (keine Incentives). Die 16 rekrutierten Personen verteilen sich über 6 Bundesländer (Baden-Württemberg, Hessen, Nordrhein-Westfalen, Rheinland-Pfalz, Schleswig-Holstein, Thüringen).

Das Sample lässt sich wie folgt beschreiben:

- Alter: 80 bis 92 Jahre,
- Geschlecht: 9 männlich, 7 weiblich,
- Wohnsituation: 13 Privatwohnung, 3 Seniorenwohnheim,
- Lebenssituation: 9 Paar, 4 alleinstehend, 3 verwitwet,
- höchster Bildungsabschluss: 5 Volks‑/Hauptschule, 5 mittlere Reife/Realschule, 3 Abitur, 1 Berufsausbildung, 2 Hochschulabschluss,
- Ehemals berufstätig: 11 ja, 5 nein.

### Durchführung und Auswertung

Im Vorfeld erhielten sämtliche Interviewten eine Aufklärung über das Gesprächsthema sowie eine schriftliche Einverständniserklärung. Die 16 Interviews wurden zwischen Oktober 2019 und März 2020 geführt. Es handelte sich um digital aufgezeichnete Einzelinterviews, in 13 Fällen mündlich-persönlich und in 3 Fällen telefonisch.

Die im Anschluss erstellten Transkripte wurden mithilfe der Software MAXQDA ausgewertet. Die Datenanalyse erfolgte auf Basis einer Inhaltsanalyse nach Mayring [26]. Im Zuge dessen wurde dicht entlang der Schwerpunkte des Leitfadens ein Kategoriensystem erstellt, das wiederholt getestet und im Laufe des Auswertungsprozesses angepasst wurde.

## Ergebnisse

Im Zuge der Auswertung ließen sich unter den Interviewten verschiedene Muster im Hinblick auf die Motive und Gratifikationen der Aneignung neuer Medien identifizieren. Zur Veranschaulichung werden im Folgenden Beispiele präsentiert, die diese Muster möglichst anschaulich widerspiegeln.

### 1) Neue Medien als Hobbyerweiterung

Seit Jahrzehnten geht Herr D. (80 Jahre) dem Hobby der Ahnenforschung nach. Nach eigener Aussage hat sich diese genealogische Leidenschaft verstärkt, seit der Lokalpolitiker in Rente ging. Lange Zeit teilte er das Hobby mit einem guten Freund, mit dem er „gemeinsam die Archive hinab[stieg]“ und sich beim „Aufdecken von Geheimnissen des Familienstammbaums“ unterstützte (I-D‑m, 11.12.2019). Infolge einer schweren Erkrankung ist der Freund jedoch nicht mehr zu Archivausflügen in der Lage, sodass Herr D. mit seinem Hobby allein blieb.

Herr D. räumt ein, er habe bereits früher von den Möglichkeiten gehört, die einem das Internet mit Blick auf die genealogische Recherche eröffnet, doch habe er sich „nie so wirklich überwinden können“, sich mit dem für ihn fremden Medium Internet zu beschäftigen (I-D-m). Da jedoch sein Freund nicht mehr zur Verfügung stand, war es „eine Mischung aus Neugier und Notwendigkeit“, die nun sein Interesse weckte (I-D-m). Vor 3 bis 4 Jahren begann Herr D., verschiedene Computerkurse zu besuchen. Anfangs seien PC und Internet für ihn eine „recht fremde Welt gewesen“, doch dann sei er immer besser zurechtgekommen (I-D-m). Auf Basis der erlernten Grundlagen setzte er die Beschäftigung mit dem Computer eigenständig fort und wandte sich den einschlägigen Portalen zur privaten Stammbaumforschung zu.

Heute sieht Herr D. seine ins Internet übergewanderte Ahnenforschung als „natürliche Weiterentwicklung“ seines Hobbys (I-D-m). Besonders die „Möglichkeiten der Vernetzung, des Austausches und der gegenseitigen Unterstützung“ von und durch andere User faszinieren ihn (I-D-m). Mittlerweile hat er sich ein reiches Kontaktnetz aufgebaut und nutzt die Vorzüge kollaborativer Recherchen. Aufgrund der „enormen Motivation und Bestärkung“, die er hierdurch empfinde, sei sein Internetkonsum „stetig gestiegen“ (I-D-m). Weil er von anderen Usern immer wieder Buch- und Weiterbildungsempfehlungen erhalten habe, sei die Entdeckung von Onlinebestellungen für ihn der nächste logische Schritt gewesen. Inzwischen verfügt er auch über einen guten Überblick über diverse Nachrichten- und Gesundheitsseiten.

### 2) Neue Medien als Hilfsnetz

Frau T. (83 Jahre) ist nicht nur aufgrund starker Arthrose und rheumatischer Beschwerden körperlich eingeschränkt, sondern seit etwa einem Jahr verwitwet. „Ich habe festgestellt, dass ich mich doch sehr stark um meinen Mann gedreht habe“, berichtet sie. „Dann steht man auf einen Schlag doch plötzlich alleine da“ (I-T‑w, 17.01.2020). In ihrem stark ländlich geprägten Wohnumfeld ist sie auf Mobilität angewiesen. Sie besitzt zwar den Führerschein, doch das Autofahren falle ihr inzwischen schwer. Frau T. schildert, vor einem Dreivierteljahr über eine Bekannte von einer lokalen ehrenamtlichen Fürsorge- und Hilfsplattform im Internet erfahren zu haben. „Das war für mich ein Grund, da mal hineinzusehen. Mein Enkelkind hat mir damals dabei geholfen und mir vieles beigebracht“ (I-T-w). Frau T. räumt ein, dass es ihr vermutlich nicht so schnell gelungen wäre, sich mit der Internetnutzung vertraut zu machen, hätte sie nicht die Möglichkeit gehabt, immer wieder Rückfragen an ihren Enkel zu stellen.

Im Laufe der Zeit hat Frau T. häufiger auf das Hilfsnetzwerk zurückgegriffen, wenn es darum ging, Laienhelfer für Einkäufe oder Unterstützung in Haushalt und Garten zu gewinnen. Mit der Zeit hat sie neue Funktionen der Plattform ausgekundschaftet. Diese ist z. B. direkt verbunden mit Informationen über Aktivitäten innerhalb der Gemeinde. „Über diese Möglichkeiten kann ich auch erfahren, was in der Nähe so los ist. Kulturelle Veranstaltungen, Feste. […] Ich habe schon Leute kennengelernt, mit denen man sich regelmäßig trifft“ (I-T-w). Auch in einem Unterportal, das sich speziell an Senioren richtet, hat Frau T. sich angemeldet, um mit Personen im gleichen Alter in Kontakt treten zu können.

Kürzlich hat sie Helfer in Anspruch genommen, die ihr weitere Kenntnisse im Umgang mit dem Internet vermittelt haben. Auch in die Nutzung einiger hilfreicher Apps zur Nachbarschaftshilfe wurde sie eingeführt und hat sich im Zuge dessen ein Smartphone angeschafft. „Ich habe den Nutzen des Internets zu schätzen gelernt“, stellt Frau T. fest (I-T-w). Inzwischen macht sie auch Gebrauch von Onlinebestellungen. Sie ist zuversichtlich, auch in den nächsten Jahren in ihrer alten Wohnung bleiben zu können.

### 3) Neue Medien als Kompensationsinstrument

Der in einem Seniorenwohnheim mit privater Wohnung lebende Herr K. (93 Jahre) vertieft sich traditionell mit großer Hingabe in die vormitttägliche Zeitungslektüre. Als jemand, der „die Welt schon immer begreifen“ wollte, ist für ihn die tägliche Auseinandersetzung mit der Tageszeitung nicht nur „Bildung und Gymnastik für den Geist“, sondern hat auch eine tagesregulierende Funktion (I-K‑m, 03.02.2020).

Für Herrn K. war es „besonders schmerzhaft“, dass sich seine Augen in den letzten Jahren beträchtlich verschlechterten (I-K-m). Trotz diverser Eingriffe ließ sich seine Sehqualität nicht mehr so herstellen, dass es für das normale Lesen einer Tageszeitung reichte. Hilfestellungen wie etwa die Verwendung einer Lupe oder das Angebot seiner Frau, ihm vorzulesen, waren nach seiner Schilderung keine „dauerhaften Lösungen“ (I-K-m).

Es war eine seiner Enkelinnen, die ihn auf den Gedanken brachte, „es doch mal mit einem iPad zu probieren“ (I-K-m). Früher, gibt Herr K. zu, hätte er sich nicht für „so ein neumodisches Zeug“ erwärmen lassen, doch nachdem ihm das Ende seiner liebgewonnenen Lektüre drohte, habe er nun „nichts mehr gesehen, was dagegen spricht“ (I-K-m). Mit Unterstützung der Enkelin ließ sich Herr K. beraten und legte sich einen Tablet-Computer zu. In den ersten Wochen, räumt er ein, sei es eine Umstellung gewesen, die Zeitung auf einem digitalen Lesegerät zu rezipieren. Doch habe er sich daran gewöhnt und sei nun „unendlich froh“, dass er sich auf das Erlernen dieses Geräts eingelassen habe. Mit dem Tablet-Computer könne er „alles beliebig größer und wieder kleiner machen“ (I-K-m).

Herr K. hat mit der Zeit begonnen, eigenständig neue Funktionen des Geräts auszuprobieren und v. a. die Nachrichtenseiten im Internet zu erkunden. Inzwischen hat er ein Abo für die Nutzung zusätzlicher Onlinenachrichtenangebote abgeschlossen. Er resümiert, der Umgang mit diesem digitalen Medium sei „zweifellos eine Bereicherung“ seines Alltags (I-K-m).

### 4) Neue Medien als Anschlussmöglichkeit

Für Frau N. (82 Jahre) war es „damals ein kleiner Schock gewesen“, als sie vor 4 Jahren erfuhr, dass ihre Familie in die USA ziehen würde (I-N‑w, 17.11.2019). Aber dann habe sie begonnen, sich mit dieser Situation zurechtzufinden und „das Beste daraus gemacht“ (I-N-w). Im Grunde wisse sie, so die frühere Sekretärin, nicht mehr genau, wie sie ursprünglich auf das Internet gekommen sei, nur dass sie von Facebook erfahren habe und über die „Möglichkeiten, über das Internet zu telefonieren, sich zu schreiben und in Kontakt zu bleiben“ I-N-w). So habe sie entschieden, mehrere Computerkurse zu besuchen, und habe sich speziell für den Umgang mit dem sozialen Netzwerk interessiert.

Frau N. räumt ein, es sei ihr anfangs v. a. mit Blick auf die Funktionsvielfalt und viele englischsprachige Begriffe „nicht leicht gefallen, in meinem fortgeschrittenen Alter damit zurechtzukommen“, doch irgendwann habe es „dann ‚Klick‘ gemacht“ (I-N-w). „Vielleicht war dieser Anstoß mit dem Umzug – so schlimm ich den auch fand – genau richtig“. Heute sagt sie von sich, sie kenne sich mit den grundlegenden Funktionen und Anwendungsmöglichkeiten des Internets aus. Besonders freue sie, dass sie über Facebook, via E‑Mail oder „manchmal sogar Skype“ regelmäßigen Kontakt zu ihrer Tochter und ihren Enkeln halten könne, sodass „sich die ganze Mühe am Ende doch gelohnt hat“ (I-N-w).

Mittlerweile reicht ihr Kontaktnetzwerk über den familiären Kreis hinaus. Irgendwann sei sie schlicht neugierig geworden und habe in Facebook über den „Tellerrand hinaus geschaut“ (I-N-w). So habe sie ein paar alte Freundinnen dort gefunden. Gelegentlich nutzt sie heute das Netzwerk auch, um sich für kulturelle Aktivitäten zu verabreden, z. B. wenn sie ins Theater geht. Das Schöne am Internet, so fasst Frau N. zusammen, sei, dass es „irgendwie ein großer Treffpunkt“ sei (I-N-w).

### 5) Neue Medien als Ausbruch aus dem Alltag

Ähnlich äußert sich Frau Q. (81 Jahre) bezüglich ihrer Erfahrungen mit dem Internet. Für sie ist das Surfen im WWW mit dem Empfinden verknüpft, „neue Ideen und Impulse zu bekommen und […] noch etwas zu erleben“ (I-Q‑w, 20.10.2019). Nachdem sie vor einigen Jahren ihren Mann verlor, merkte sie, „wie einsam es um mich herum geworden ist“. Gute Freunde habe sie nicht besessen, weshalb sie sich rasch isoliert gefühlt habe. „Das Schlimmste war dieser eintönige Alltag“, schildert sie (I-Q-w).

Frau Q. stellt dar, dass der Anstoß für ihre Beschäftigung mit dem Internet in erster Linie „ganz praktisch“ damit zu tun gehabt habe, als „körperlich eingeschränkte Witwe“ ihren Haushalt zu organisieren (I-Q-w). Da sie verstärkt von Onlinebestellungen (z. B. über Discounterlieferservices) Gebrauch machen wollte, habe sie begonnen, Computerkurse zu belegen.

Mit der Zeit habe sie jedoch das Internet „in einem ganz großen Ausmaß“ für sich entdeckt. So habe sie sich in mehreren Online-Communities angemeldet und dabei festgestellt, dass man „mit Personen schreiben [kann], denen es ähnlich geht wie einem selbst, man kann sich gegenseitig ein bisschen Mut zusprechen, man ist niemals ganz allein“. Diese Erkenntnis, so Frau Q., habe ihr über den Verlust ihres Mannes „ein Stück weit [hinweg]geholfen“ (I-Q-w).

Weitere Angebote, die Frau Q. inzwischen nutzt, sind Videoportale und Mediatheken, auf denen sie immer wieder „anregende Information und Unterhaltung“ finde. Heute sei das morgendliche Frühstücken am Computer ein „tägliches Ritual“ geworden. Es vermittelt ihr das Gefühl, „dass ich nicht wirklich so allein bin, sondern mit der ganzen Welt verbunden […], dass man noch ausbrechen kann, wenn man das möchte“ (I-Q-w).

### Weiterführende Analyse des Samples

Jenseits der dargestellten Einzelbeispiele zeigt sich für das Gesamtsample, dass nach Aussage der Interviewten mehrere Motive besonders vorrangig und ausschlaggebend waren, um den Umgang mit dem jeweils ausgewählten digitalen Medium zu erlernen (in einigen Fällen wurden mehrere primäre Beweggründe genannt). So wurde in 8 Fällen eine deutlich verbesserte, manchmal auch aufgrund von Umzügen erforderliche Kontakt- und Kommunikationsmöglichkeit mit anderen Familienmitgliedern und/oder Freunden genannt. In 7 Fällen wurden physische Einschränkungen (Erkrankungen, Gebrechlichkeit) bzw. altersbedingt nachlassende Mobilität angesprochen, die durch Erlernen des Mediums zumindest in Teilen kompensiert werden sollten (z. B. indem Einkäufe online erledigt werden können). Insgesamt 6 Interviewte brachten als Aneignungsmotiv v. a. Interessen- und Hobbyzusammenhänge vor, während in 5 Fällen eine soziale Um- oder Neuorientierung aufgrund des Verlustes von Kontakten (insbesondere Verlust des Partners durch Tod oder Trennung) stattfand. Hier wurde nicht nur Interesse an neuen Kontakten positiv angeführt, sondern auch eine Sorge vor Vereinsamung teils offen thematisiert.

Obgleich die Berichte der meisten Interviewten eine vergleichsweise rasche, zielgerichtete und gelingende Medienaneignung vermuten lassen, wurden von den Befragten auch erlebte Hürden und Barrieren angesprochen. Im Zusammenhang mit der Nutzung des Internets wurde etwa darüber berichtet, dass die Nichtlinearität und Multioptionalität des Mediums gerade zu Anfang für Überforderung gesorgt hätten. In ähnlicher Weise wurde die Notwendigkeit zur Beschäftigung mit fremdsprachlichen Begriffen als Herausforderung und Umstellung erlebt. Ein Teil der Befragten nahm beim Erlernen einen hohen kognitiven Aufwand und starke Beanspruchung wahr, sodass immer wieder pausiert werden musste. Zudem wurde davon gesprochen, dass erst einmal viel an Wissen aufgenommen und Kenntnisse eingeübt werden mussten, was eine intensive, manchmal belastende Beschäftigung mit dem digitalen Medium erforderte. Was es den Befragten nach eigener Darstellung teilweise erleichtert hat, die Nutzung zu erlernen, war zunächst eine Konzentration auf wenige bestimmte Funktionen, wohingegen andere Nutzungsmöglichkeiten erst einmal ausgeblendet wurden.

## Diskussion

Die 16 interviewten Personen haben sich im fortgeschrittenen Lebensalter digitale Medien zielgerichtet angeeignet. Bei der Beschäftigung mit PC, Tablet-Computer, Smartphone und Internet wurde zu Anfang von Hilfestellungen wie Computerkursen oder Unterstützung durch Enkelkinder Gebrauch gemacht, bis selbstständig weitere Nutzungsmöglichkeiten erkundet wurden. Trotz anfänglicher Hürden und Herausforderungen bei der Aneignung ist es den Interviewten gelungen, das neue Medium sinnstiftend in ihren Alltag zu integrieren.

Zugleich ist im Zuge der Interviews deutlich geworden, dass solche Aneignungsprozesse weniger aus spontanen Launen, Neugier, Experimentierfreude oder Gruppendynamiken heraus entstehen, sondern eher durch gravierende Veränderungen der Lebensumstände angestoßen werden [29, 30]. In vielen Fällen ist ein Einbruch des bisherigen sozialen Gefüges oder zumindest eine problematische Veränderung des Alltags zu verzeichnen, die eine Neuorientierung mithilfe neuer Medien lohnenswert erscheinen lässt. Der in den Interviews oft zu konstatierende Ausgangspunkt für die Beschäftigung mit einem digitalen Medium liegt deshalb zunächst oft in einem Anpassungs- bzw. Kompensationsbedürfnis begründet. Dies kann möglicherweise als spezifisches Momentum der Medienaneignung im höheren Lebensalter aufgefasst werden und wird von mehreren Arbeiten gestützt [14–16, 20, 32, 36]. Gonser führt aus, dass irreversible Veränderungen von Körperfunktionen, sozialen Kontakten und Wohnsituationen „nicht selten zu einer veränderten Praxis des Medienhandelns [führen], die den im Lebenslauf habitualisierten Mediennutzungsmustern zuwiderlaufen“ [12, S. 77].

Wie Schorb [35] beschreibt, nutzen ältere Menschen Medien nicht nur zur Überwindung von Einsamkeit oder Mobilitätseinschränkungen, sondern sie treten diesen mit alters- und persönlichkeitsspezifischen Bedürfnissen und Erwartungen gegenüber. Ähnliches gilt für die Aneignung neuer Medien. Eine zentrale mit dem Alter verknüpfte Aufgabe ist die Gestaltung und Bewältigung der Anforderungen des alltäglichen Lebens. Verspricht ein Medium hier „zur Bearbeitung anstehender Entwicklungsaufgaben oder aktueller Problemlagen“ [35, S. 325] konkrete Nutzungspotenziale, ist ein Aufgreifen vor dem Hintergrund eigener biografischer Vorerfahrungen und Interessen attraktiv.

Die meisten der Interviewten haben sich ein internetfähiges Medium erschlossen, weil sie bedürfnisorientiert ganz gezielte Funktionen nutzen wollten. Sie sahen es als erforderlich an, zur Unterstützung im Alltag bestimmte Kompetenzen zu erwerben, verharrten dann aber erst einmal bei den anvisierten Applikationen. Dies spiegelt sich u. a. in einer internationalen Repräsentativbefragung älterer Internetanwender wider [8]. Erst mit einiger Verzögerung haben sich bei den Befragten neue Anwendungsgebiete ergeben; als Folge davon konnten neue Kommunikationsräume, Gesprächs- bzw. Interessenthemen erschlossen werden.

### Limitationen

Ziel der Studie war es, Aneignungsprozesse digitaler Medien bei älteren Menschen explorierend zu beleuchten, insbesondere mit Blick auf Anlässe und Motive. Eine Stärke der Arbeit ist in der untersuchten Zielgruppe zu sehen, da es insbesondere für hochaltrige Menschen bislang an entsprechenden Forschungsarbeiten mangelt. Hinsichtlich des gewählten qualitativen Zugangs besteht ein Vorteil zum einen darin, dass ältere Mediennutzer möglichst unvoreingenommen betrachtet und, im Unterschied zu geschlossenen quantitativen Befragungen, neue Aspekte beleuchtet werden konnten. Zum anderen konnte über die Interviews die Lebenswirklichkeit der Befragten eingefangen und, damit einhergehend, der Nutzen einer Integration neuer Medien in den eigenen Alltag besser nachvollzogen werden.

Dennoch ist auch auf Schwachstellen der Studie hinzuweisen. Trotz der heterogenen Zusammensetzung des Samples handelt es sich um eine vergleichsweise kleine und selektive Stichprobe, die keine Repräsentativität für die Grundgesamtheit hochaltriger Internetnutzer beanspruchen kann. Hinzu kommt, dass es sich bei der Stichprobe um überwiegend gesunde und relativ selbstbestimmt agierende Senioren handelte. Überdies stellte die Rekrutierung geeigneter Studienteilnehmer in der Altersgruppe ab 80 Jahre eine Herausforderung für die Autoren dar. Daher ist eine Aussage schwierig zu treffen, inwieweit mit dem letztlich gewonnenen Sample eine ausreichende theoretische Sättigung erreicht werden konnte.

Im Zuge der Studie haben ältere Personen aus ihrer Erinnerung berichtet, wie es zur Aneignung digitaler Medien gekommen ist. Inwiefern dies tatsächlichen Gegebenheiten entspricht, kann nicht überprüft werden. Hierzu wären komplexere teilnehmende Beobachtungen notwendig, um den Vorgang der Medienaneignung im höheren Lebensalter genauer zu beleuchten. Zwar wurde bei der Rekrutierung sichergestellt, dass bei den Befragten bis zum Zeitpunkt der betrachteten Aneignung eines digitalen Mediums keine Berührungspunkte mit Medien wie Computer, Internet oder Smartphone bestanden; dennoch konnte im Zuge der Interviews nicht vollständig und erschöpfend auf die technische Ausstattung im Haushalt der Befragten oder auf in der Vergangenheit erworbene technische Fähigkeiten eingegangen werden.

Nicht zuletzt ist darauf hinzuweisen, dass die Studie Teilnehmer fokussierte, die in den vergangenen Jahren ein digitales Medium erfolgreich erschlossen haben. Demgegenüber sind solche Personen nicht näher betrachtet worden, die versucht haben, den Umgang mit einem neuen Medium zu erlernen, aber aufgrund von zu großen subjektiven Hürden beim Versuch der Aneignung gescheitert sind [11, 12].

## Ausblick

Für ältere Menschen können digitale Medien gewinnbringende Nutzungspotenziale offerieren, die einen Mehrwert im Alltag eröffnen können. So können digitale Medien in Anlehnung an Lehr ein „Tor zur Welt“ sein, das den Rückgang sozialer Kontakte kompensiert; sie können aber auch als „Ratgeber […] zur Lösung alterstypischer Lebensaufgaben“ dabei behilflich sein, ein neues „Netz der Gewohnheiten“ zu errichten, indem z. B. Kommunikation im familiären Raum über digitale Lösungen sichergestellt wird [23, S. 132 f.]. Ebenso können neue Medien als „Stimulationsquelle“ fungieren [23, S. 132 f.].

Bislang ist nur wenig über Vorgänge und Motive der Medienhinwendung und -aneignung bei älteren Menschen bekannt, insbesondere gilt dies für Personen im hochaltrigen Segment. Die Interviewstudie hat ältere Menschen beleuchtet, die unter der Voraussetzung von Motiven, die mit der Aussicht klarer Mehrwerte korrespondieren, ein durchaus hohes Maß an Anpassungs- und Lernbereitschaft in Bezug auf den Umgang mit neuen Medien zeigen. Auch liefern die Interviews Hinweise, dass Aneignungsprozesse bei Vorhandensein solch intrinsischer Beweggründe zielstrebiger und effektiver ablaufen können.

Insgesamt erscheinen daher vor dem Hintergrund der Befunde motivationsorientierte Ansätze als wichtige Ergänzung der bestehenden Alter(n)smedienforschung, um generationsspezifische Medienpraxiskulturen und Voraussetzungen für die Medienaneignung bzw. -gratifikation im höheren Lebensalter zu erklären [12]. Dabei werden unter Berücksichtigung von sozialen und psychologischen Faktoren des Alter(n)s v. a. individuelle Nutzungsmotivationen betrachtet, die z. B. mit einschneidenden Lebensereignissen einhergehen können. Weitere Forschung in diesem Bereich erscheint vielversprechend und sollte erkunden, inwiefern die Aneignung neuer Medien mit Bewältigungs- und Umgangsstrategien im höheren Lebensalter korrespondiert [36]. Stärker als bei jüngeren Altersgruppen sind mit Blick auf altersspezifische Motiv- und Bedürfnislagen gegenüber Medienangeboten zudem biografische Kontexte zu integrieren.

Nicht zuletzt ist von Bedeutung, dass gerade in der Gruppe der Hochaltrigen die Zahl der Personen kontinuierlich zunimmt, die in teilstationären oder stationären Einrichtungen lebt. Folglich wird es für künftige Arbeiten auch darauf ankommen, ältere Mediennutzer jenseits der privaten häuslichen Lebenssituation in den Blick zu nehmen, deren Möglichkeiten, Bedürfnisse und Interessen sich von dem in dieser Untersuchung rekrutierten Sample unterscheiden.

## Fazit für die Praxis

- Es ist davon auszugehen, dass ältere Menschen bei Vorhandensein konkreter Nutzungsmotive den Umgang mit neuen Medien als sinnhaft empfinden und erlernen. Dabei sind biologische, psychische und soziale Zusammenhänge sowie Bewältigungs- und Umgangsweisen mit dem Alter(n) bedeutsam.
- Die Interviewten zeigten eine beträchtliche Anpassungs- und Lernfähigkeit bei der Beschäftigung mit PC, Tablet-Computer und Internet. In vielen Gesprächen fällt auf, dass Aneignungsprozesse durch gravierende Veränderungen der Lebensumstände angestoßen wurden.
- Zudem haben die meisten der Interviewten sich ein internetfähiges Medium erschlossen, weil sie ganz gezielte Funktionen nutzen wollten. Mit der Zeit sind neue Möglichkeiten der Onlinenutzung entdeckt worden.
